# The Application of Similar Image Retrieval in Electronic Commerce

**DOI:** 10.1155/2014/579401

**Published:** 2014-04-22

**Authors:** YuPing Hu, Hua Yin, Dezhi Han, Fei Yu

**Affiliations:** ^1^School of Information, Guangdong University of Finance & Economics, Guangzhou 510320, China; ^2^College of Information Engineering, Shanghai Maritime University, Shanghai 20130, China

## Abstract

Traditional online shopping platform (OSP), which searches product information by keywords, faces three problems: indirect search mode, large search space, and inaccuracy in search results. For solving these problems, we discuss and research the application of similar image retrieval in electronic commerce. Aiming at improving the network customers' experience and providing merchants with the accuracy of advertising, we design a reasonable and extensive electronic commerce application system, which includes three subsystems: image search display subsystem, image search subsystem, and product information collecting subsystem. This system can provide seamless connection between information platform and OSP, on which consumers can automatically and directly search similar images according to the pictures from information platform. At the same time, it can be used to provide accuracy of internet marketing for enterprises. The experiment shows the efficiency of constructing the system.

## 1. Introduction

With the development of informatization in the small- and medium-sized enterprises, online marketing becomes a good choice for these enterprises [1, 2]. Among all kinds of channels of online marketing, the small- and medium-sized enterprises are more prone to choose the third party B2B online marketing platform and search engine, which provides the packaged IT information and commerce services. Research from iResearch shows [3] the network marketing costs in China will be up to 8% in 2008 from 0.5% in 2002. And it predicts that the proportion will rise continually and reach to 11.5% in 2013. 

Early OSP generally uses the keyword searching mode. It is difficult to describe the products with simple word because of their diverse styles and rich color. Customers cannot search the needed products directly. Meanwhile, products update quickly, and labeling so many products artificially is a tremendous work. The inconsistency of description between customers and suppliers leads to inaccuracy in search results and large search space; then it will consume too much time for customers. Therefore, the existing problems, such as indirect search, inaccuracy in search results, and long shopping time from the keywords searching mode, will influence customer experience on online shopping. On the other hand, from the trend of internet developing, 80% of customers will browse information on internet, but among them only 28% will take part in online shopping. How to lead the internet users from browsing information to online shopping is a key problem for electronic commerce advertisers. Traditional advertisement injects advertisements in the information platform, in which purchasing pages are linked by advertisements clicking. This method will lead to low clicks ratio and low actual purchase ratio for the irrelevance of injecting advertisements and information and the false clicks of customers. Therefore, despite electronic commerce customer or electronic commerce suppliers, online shopping needs to combine with visual, interactional, and functional new image searching technology.

Starting with solving the problem of traditional online marketing, we research the application of similar image retrieval in electronic commerce and promote the corresponding application system design proposal. This system connects information platform with purchase platform. Customer can automatically search similar image according to their favorite pictures from information platform by this system. Eventually it will increase the purchase ratio of customers. The system can also automatically collect the product information from the other online shopping platform, provide direct similar image retrieval for customers, and display the search results with the customized way.

## 2. Similar Image Retrieval and Application Analysis

**Figure 1:**
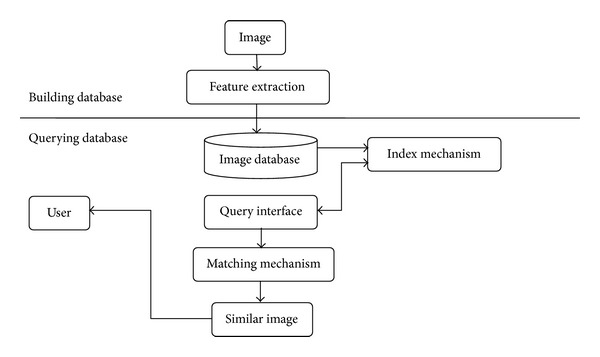
The basic framework of similar image retrieval system.

Similar image retrieval [4–6] is a new vision image searching technology used to solve the above problems. This technology searches images according to their content and context environment. Firstly, some objective image features are extracted automatically by computer, such as color, grain, shape, and spatial relationship. Then the image is indexed in database based on these features. According to the sample image, the users retrieve image in database by some similarity measurements, and the system returns similar results to users. This technology provides a new search mode—searching by image. The mode of searching by image will help users find the same or similar image in plenty of internet images. It relieves the embarrassment of customers, when they face some image, which is difficult to describe by keywords, or they do not want search by keywords. The visualization of searching by image satisfies the customers with higher searching requirements, helps customers find product information more conveniently, and elevates the online shopping experience. The vision image searching results by themselves can make customers buy the product more quickly, and visual display can increase the click-pursue turnover rate.  Figure 1 is the basic framework of similar image retrieval system.

Similar image retrieval system includes three components: feature extraction, effective retrieval, and user interface.

### 2.1. Feature Extraction

Image features include primitive features and semantic features. The primitive features of image, which are quantitative, include the color, shape, texture, and spatial relationship. The semantic features of image, which describe the image content, are qualitative. They are extracted by artificial way or human-computer interaction.

### 2.2. Effective Retrieval

Effective retrieval includes indexing and matching mechanism. Currently, the general index models include *k*-d tree, *R*-tree and variety *R* ^+^ tree, R* tree, VA-File, and so on [7]. Commonly used matching mechanisms (similarity measurement methods) include Euclidean distance and histogram interaction distance. Reasonable indexing and matching mechanism is necessary for image retrieval.

### 2.3. User Interface

The searching modes provided by user interface include query by keyword, query by sketching, query by example, browsing by categories, feature selection and weighting, and relevance feedback. Firstly, user delivers the sample image by query interface;, then system computes similar measurement according to the image features. At last, system returns to user the query results according to the rank of similarity.

In the application of similar image retrieval, some research institutions have distributed their prototype system on the internet. Among them, there are QBIC [8] developed by IBM, VisualSEEK [9] developed by Columbia University, and Virage [10] developed by Virage. Most of these systems are prototype systems or image retrieval systems facing some professional field, such as digital library and face reorganization. It is a new application field to apply similar image retrieval to electronic commerce for image searching. And it has considerable commercial value. Its application prospect is represented in three aspects. (1) On the trend of internet electronic commerce, pictures are the best medium to exhibit product and make transactions success. Its visual search mode will become the main entry of online shopping. Searching by image, which is functional and visual, satisfies users with higher searching requirements, provides convenience to find product information, and elevates the experience of electronic commerce online shopping. (2) Traditional advertisements on the website have too long linkage from clicking advertisements to purchasing. The main function of them is to exhibit the product brand. The actual commodity buying rate is very low. Similar image retrieval, which is actively done by user, can help user pursue the commodity more quickly. The visual exhibition can improve the click-purchase rate. B2C electronic commerce supplier can realize the accuracy in sales by connecting their own commodities according to some behavior index such as similarity and focus degree of searching results. (3) In mobile electronic commerce, it is inconvenient to input words in mobile phone. It makes replacing word with image be the main information carrier of mobile terminal, which leads to increasing delivering picture information requirements of users. But the channel of picture delivering between mobile terminal and pc platform has not opened. How to make user transfer picture information by mobile terminal to electronic commerce platform becomes an urgent problem in the linkage of electronic commerce transaction. Similar image retrieval helps open the information transformation channel between mobile terminal and electronic commerce platform for its characteristics of searching by image. Users can upload image in real time, search, and then buy the commodity according to the searching results. It thoroughly solved the above problem. Huge commodity image database includes information coming from all kinds of C2C and B2C platform. It is a natural mobile electronic commerce platform.

Facing various commodities, customers are often interested in looking for more similar others to compare with their favorite one. When most of network users browse all kinds of information website, they may be interested in the goods in the pictures and wish to find similar clothing on the internet. But current searching engine cannot help customer realize it. The blank market has huge commercial value. Applying similar image retrieval to electronic commerce can fill in the blank market and provide a convenient way to search commodities which are difficult to describe by internet users. At the same time, it will provide a good network marketing platform. This special searching mode will attract plenty of users to use this platform. If we use the most acceptable CPS marketing payment mode, it is predicted that plenty of enterprises will adapt this technology to help introduce their own products. If we compute the network sales according to 30 billion yuan in 2009, 0.01 percent of clothing online shopping sales use the platform adapting similar image retrieval to sale, and provided we receive 10% commission according to the average proportion of CPS in the clothing market, this project will realize sales to 308 hundred thousand yuan. The essence of similar image retrieval makes it be the controller of future user traffic. It satisfies users with the visual requirements for image searching. And the accuracy in commerce mode extremely improves the electronic commerce advertisement recourse. Similar image retrieval pushes the development of electronic commerce in online shopping experience or in the electronic commerce marketing. At last, it receives double win between customers and enterprises.

There are limited references about the application of similar image retrieval to electronic commerce to search commodity image. Lin et al. [11] promote an integrated multiple feature and multistage commodity image retrieval method, which integrated various primitive features such as color, texture, and shape. For improving the searching speed, it firstly uses texture to do the primary searching and then does the integrated searching using various features. In addition, for improving the searching results, they promote a multifeature dynamic weight allocating method, which dynamically allocates weights to different types of primitive features according to the different image resolution. In 2006, Like, an online shopping website in America, firstly applies similar image retrieval to online shopping and provides visual commodity searching mode [12]. The visual searching experience in Like is very good, and customer can quickly find many highly similar commodities. In China, it is a test phrase to apply similar image retrieval to online shopping. Taotaosou, an image searching and online shopping website invested by Alibaba Corporation, is the only practical product [13]. Although this website has collected plenty of merchant and image information and users can find similar image by uploading or using a sample picture, during the process of using it, the searching needs the user to predefine the classification of commodities and the target area of commodity image. There are big differences between searching results and the searching results sometimes are not accurate. The searching process to find target product consumes too much time.

## 3. Electronic Commerce Platform Design with Similar Image Retrieval System

**Figure 2:**
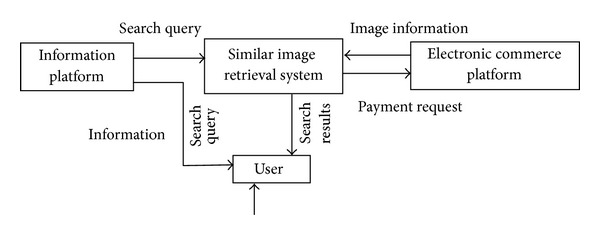
The relationship of three systems.

Similar image retrieval system is a medium between information platform and online shopping platform for realizing their seamless connection, reinforcing customer experience, and introducing products to increase the product pursue rate. The relationship among them is shown in Figure 2.

There are three implementation modes of similar image searching system: combination, independent, and embedded. The combination mode needs to add information modular and payment modular in system to make the system provide the browsing service and online safety payment function. The independent mode, as an independent system, needs to separately provide an interface between information platform and all kinds of online shopping platforms, while embedded mode uses the idea of plugin to embed into information platform. The overall system framework is shown inFigure 3.

We plan to design an integrated multifeature searching algorithm based on interested regions. As the direct mapping of the objective world, images are huge and have abundant objective expressiveness. Integrated multifeature searching based on interested region is an ensemble technology, including image feature extraction, image feature matching, image data modeling, high dimensional index, relevance feedback, and searching performance evaluation. We plan to use Harris operator to extract interested point, because Harris operator [13] is stably facing rotation and translation and the change of brightness. In feature extraction, we plan to combine color feature and space feature mentioned in international standard MPEG-7 of multimedia retrieval. Although global color feature is easy to compute and provides good discrimination, it is incapable of handling color query related to the space. The experiments have proved that the proportion of precision between color, space feature and texture, and shape feature is 2.5 : 1. We will use the weighted histogram interaction and L1 distance as the similarity measurement. The image feature, used in similar matching, can be got by interacting with users or extracting from the image. User provides the query to system, and system returns searching results according to the query. At the same time, user can use the relevance feedback of searching results to improve searching results. It is inevitable to face high dimensional feature for using integrated multifeature searching. We solve the high dimensional index problem by dimension reduction. We utilize the characteristic of manifold, which can directly find implicit low dimensional nonlinear data structure in observation space to do the nonlinear dimension reduction.

System components are showed as below.

**Figure 4:**
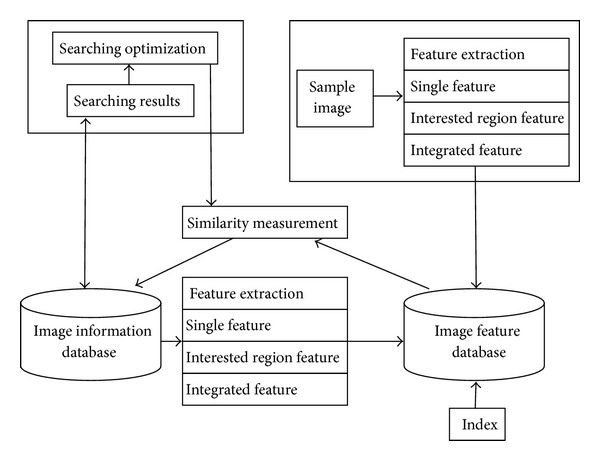
Image retrieval subsystem.

**Figure 5:**
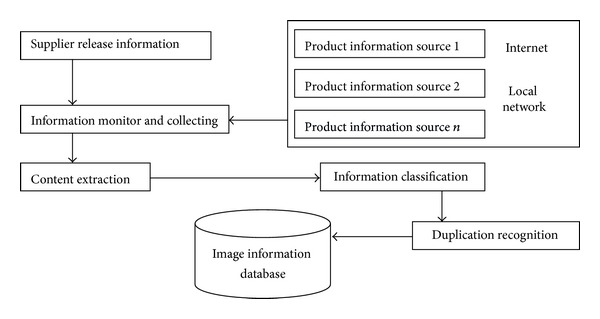
Product information collecting subsystem.

1. Image retrieval display subsystem: the essence of this subsystem is a website to provide information browsing, searching query, and costumed tool and searching results display service. For conforming to the idea of “high cohesion, low coupling,” the website is designed into 3 tiers: display layer (UI), business layer (BLL), and data access (DAL).
2. Image retrieval subsystem: the main task of it is to execute image retrieval algorithm. It includes three parts. The first one is to extract the commodity image features and then store them into image feature database for future query. The second one is to get query order from image retrieval display subsystem, execute searching algorithm, and then return the searching results. The last one is to optimize the searching results and feedback to display subsystem. The framework of image retrieval system is different from traditional retrieval system based on text. Because the image is indexed by its visual feature not text description, the query will compute the similarity according to the image visual features. Users query by choosing representative sample image, and then system will search similar image with the sample image and return to user according to the rank of similarity. Image feature can be gotten not only through interaction with user but also from the image itself, and then are used for similar matching. User and system are interactive. User can query to system, system can return query result according to query requirement, and, meanwhile, user can improve the searching results by relevance feedback.  Figure 4 shows the structure of image searching subsystem.
3. Product information collecting subsystem: the main task of this subsystem is to collect the product information in C2C and B2C platform, including product picture, product name, product price, product introduction, and relevant link. This product information with the product information released by suppliers is sent to image database for future requirements of image retrieval. Before their entering the database, system will categorize them and recognize and throw away the duplicate image. Figure 5 gives the structure of product information collection subsystem.

The advantage of above design is as follows: (1) clear modular structure and (2) independent image retrieval algorithm process subsystem, which is convenient in optimizing and elevating system performance.

## 4. System Implementation and Test

The system implementation adapts J2EE, a widely used and gradually mature technology, as the technical framework. The system is developed by the tool below:

 S oftware Programming: Myeclipse JDK
 Interface programming: Macromedia Dreamweaver MX
 Database: NAVICAT Mysql
 Web container: Tomcat 6.0
 Language programming package: JDK1.6.

The functions of the platform have three characteristics. (1) Searching similar product image according to information image: customers need not use keyword to describe the clothing information. The only thing they need to do is to click their favorite fashion information picture; then they will get similar clothing pictures and the system will automatically link with the purchasing site of this clothing. (2) Automatically collecting and classifying clothing picture information: system can automatically collect all kinds of clothing picture information on internet and then classify and store them for future searching. (3) Optimizing the search results: it can optimize the rank of search results according to the requirements of enterprise and the popular degree of product, which will help enterprise to promote their products.

Evaluating an image retrieval method needs to choose a standard image dataset as the test platform. This image dataset must be reprehensive and large. The image database in our system includes 1200 images, which includes three types of Chinese clothing: full dress, daily clothing, and home clothing; three types of menswear: sports style, business dress style, and fashion style; three types of women's clothing: sports style, romantic style, and city dress style; three types of children's clothing: sports style, fashion lovely style, and adult style. Each type includes 100 similar images, which is of arbitrary size and less than 50 k. Too large image will influence the speed of image entering database. The format of image is bmp.

The general two criterions of evaluating searching performance, efficiency and efficacy, separately represent searching speed and success rate of searching and querying similar image. The searching efficiency usually uses the system response time to evaluate. Recall and precision, which are used in content-based image retrieval, are standard evaluation methods of information retrieval success rate. Among them, recall [14] is used to test the capability of searching relevant document. On the contrary, precision [15] is used to test the capability of excluding the irrelevant document, They can be defined by the formula below:

(1) recall=Number  of  relevant  searching  imagesNumber  of  all  relevant  images=AA+C,precision=Number  of  relevant  searching  images  Number  of  all  searching  images=AA+B.

In the formula (1), *A* represents the number of relevant searching images, *C* represents the number of relevant missing images, *B* represents the number of irrelevant searching images.

For testing the performance of the system, we use the chosen standard image dataset to test the system's performance index. About the system efficiency, experiments results show the following. (1) The average searching response time (the waiting of user wait for the searching results) is 2.24 s, no more than 5 s. (2) The average purchasing link time is 1.24 s, no more than 2 s. About the system efficacy, average search recall is 81.35%, and the smallest is 75.2%; the average precision is 83.3%, and the smallest is 76.4%. The experiment results show the effect of the system.

## 5. Conclusion

The online marketing platform based on similar image retrieval connects information platform with purchase platform. In this platform, customers can search similar image according to their favorite picture, which will increase the purchase rate of customers. This platform can also automatically collect the product information from the other online shopping platform, provide the direct similar image searching mode, and display searching results by the customized way. The target market of this proposal firstly faces the clothing field, and then it can be used in the other sales field, such as electrical equipment business and construction business. The promotion of this system can accelerate the development of electronic commerce. So it has a broad application foreground.

## Figures and Tables

**Figure 3:**
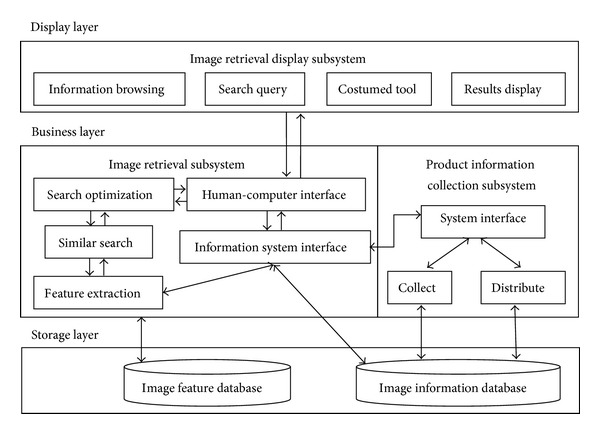
The overall system structural diagram.
